# Evaluation of the Presence of Bacterial and Viral Agents in the Semen of Infertile Men: A Systematic and Meta-Analysis Review Study

**DOI:** 10.3389/fmed.2022.835254

**Published:** 2022-05-04

**Authors:** Mehrdad Gholami, Mahmood Moosazadeh, Mohammad Reza Haghshenash, Hamed Jafarpour, Tahoora Mousavi

**Affiliations:** ^1^Department of Microbiology and Virology, Faculty of Medicine, Mazandaran University of Medical Sciences, Sari, Iran; ^2^Molecular and Cell Biology Research Center (MCBRC), Hemoglobinopathy Institute, Mazandaran University of Medical Sciences, Sari, Iran; ^3^Gastrointestinal Cancer Research Center, Non-communicable Diseases Institute, Mazandaran University of Medical Sciences, Sari, Iran; ^4^Faculty of Medicine, University of Medical Sciences, Sari, Iran; ^5^Medical Sciences Technologies, Molecular and Cell Biology Research Center (MCBRC), Faculty of Medicine, Mazandaran University of Medical Sciences, Sari, Iran

**Keywords:** bacteria, virus, infection, semen, infertility, Iran

## Abstract

**Objectives:**

Infections in the male genitourinary system with bacterial and viral agents may play a significant role in male infertility. These agents usually infect the urethra, seminal vesicles, prostate, epididymis, vas deferens, and testes retrograde through the reproductive system. A meta-analysis review study was performed to evaluate the presence of bacterial and viral agents in the semen of infertile men and its correlation with infertility.

**Methods:**

Relevant cross-sectional and/or case-control studies were found by an online review of national and international databases (Web of Science, PubMed, Scopus, Science Direct, and Google scholar), and suitable studies were selected. A checklist determined the qualities of all studies. Heterogeneity assay among the primary studies was evaluated by Cochran’s *Q* test and I^2^ index (significance level 50%). A statistical analysis was conducted using the Comprehensive Stata ver. 14 package (StataCorp, College Station, TX, United States).

**Results:**

Seventy-two studies were included in this meta-analysis. Publication bias was compared with Egger’s test, and the impact of each research on overall estimate was evaluated by sensitivity analysis. In 56 studies, the rate of bacterial infections in the semen of infertile men was 12% [95% confidence interval (CI): 10–13]. Also, in 26 case-control studies, the association of infertility in men with bacterial infections was evaluated. The results show that the odds ratio of infertility in men exposed to bacterial infections is 3.31 times higher than that in non-infected men (95% CI: 2.60–4.23). Besides, in 9 studies that examined the prevalence of human papillomavirus (HPV), herpes simplex virus 1 (HSV1), herpes simplex virus 2 (HSV2), and herpes simplex virus 1-2 (HSV1-2) in infertile men, the frequency of these viruses was 15% (95% CI: 9–21). In 6 case-control studies, the association between human cytomegalovirus (HCMV), Cytomegalovirus (CMV), and HPV and male infertility was evaluated. The chance of male infertility due to exposure to these viruses was 2.24 times higher than those without exposure to these viruses (CI 95%: 1.9–4.52). The results show that the chance of infertility in men exposed to bacteria was significantly higher than that in the uninfected population.

**Conclusion:**

This meta-analysis showed that viral and bacterial infections are a risk factor and could impair male fertility potential. Moreover, our study supports the hypothesis that bacterial and viral infections of the genital tract correlate positively with impairment of sperm quality in the male population.

## Introduction

Infertility refers to the inability to have a baby and pregnancy after at least 1 year of marriage and attempts of pregnancy without contraceptives. Causes of infertility can refer to women, men, or both (1). According to WHO studies, about 50–60% of cases are of men. In 10%, the cause of infertility is unclear, that is, a couple’s examination does not indicate a pathological problem, but the cause of infertility is unclear (2). Major causes in men include genital injuries, semen infections, testis problems, genital tracts, genital glands, varicocele, genital tract obstruction, endocrine, and metabolic diseases (3). One of the essential reasons for male infertility is semen and genital tract infections. Male urinary tract infection is one of the most important causes of male infertility. As many as 8–35% of infertility cases worldwide are due to genitourinary tract infections (4). Although the mechanism and physiopathology of infections in infertility are not fully understood, viral and bacterial infections appear to cause semen or sperm abnormalities and morphological changes in sperm, and reduce motility directly, thus reducing fertility (5). It can also indirectly cause infection, testicular damage, and inflammation and, subsequently, stimulate the immune system against intrinsic antigens associated with leukocytospermia, all of which can cause male infertility (6). The presence of leukocytes in the semen (pivotal sperm) can play an essential role in reducing the qualitative parameters of the ejaculatory fluid and, on the other hand, is a good sign in determining genital infection. Some studies suggest that leukocytes in the semen indicate infection and that those with urethritis have more leukocytes in their semen (7).

According to WHO criteria, a concentration of more than 10,00,000 leukocytes per ml of semen is called leukocytopenia. Meanwhile, polymorphonuclear leukocytes make up 50–60% and macrophages 20–30% of semen-positive peroxidase leukocytes. Leukocytes are one of the most important sources of reactive oxygen species in semen (8). How these cells enter the seminiferous tubules is not well understood. Still, studies have shown that, as a result of infection, tight junctions between Sertoli cells are destroyed, or their resistance is reduced, and leukocytes invade the seminiferous tubules (9). Since the cytoplasm of a mature sperm is low, and the concentration of ROS-destroying antioxidants is low in sperm cells, sperm cells are more susceptible to oxidative stress than any other cell (10). Also, because the sperm membrane contains large amounts of unsaturated fatty acids, it is highly vulnerable to oxidative stress.

Furthermore, due to the specific form of sperm, intracellular antioxidant enzymes cannot protect the plasma membrane surrounding the acrosome and tail (11). Among the common bacterial species, male genitourinary tract infections are caused by *Streptococcus pyogenes*, enterococci, *Escherichia coli*, coagulase positive, and negative *staphylococci* bacteria (12). If the infection destroys the blood-testicular barrier, it results in the formation of anti-sperm antibody exposure levels that are detectable in the serum and semen (13). However, it is not yet known whether serum antibodies significantly affect patients’ fertility. However, anti-sperm antibodies in semen can impair sperm function. Some bacteria also cause sperm cells to immobilize by adhesion or agglutination, which depends on the density of bacteria in the semen. *Escherichia coli* and *Chlamydia trachomatis* cause sperm agglutination, and, on the other hand, the attachment of bacteria to the sperm cell membrane results in reduced sperm attachment to the ovum (14). Some leading causes of chronic sperm viral infections are human immunodeficiency virus (HIV), hepatitis B virus (HBV), and hepatitis C virus (HCV). In particular, the role of HIV infection in chronic lower genital inflammation, sperm infection, and fertility decline is significant. Recent studies show that the presence of HBV or HCV in semen adversely affects sperm parameters and, in particular, reduces sperm motility. Also, seminal viral infection is associated with increased frequency of sperm abnormalities and DNA damage (4). According to importance of genital infections in male infertility, therefore, this study will be designed to survey viral and bacterial agents in semen of infertile men. Therefore, this study will be designed to survey viral and bacterial agents in semen of infertile men. This is a systematic review and meta-analysis of various bodies of literature in this field, so it may be possible to obtain a single result from different studies and clarify the role of viruses and bacteria in male infertility.

## Methods

Preferred Reporting Items for Systematic Review and Meta-Analysis Protocols (PRISMA-P) described the present meta-analysis statement and the guidelines of the Meta-analysis of Observational Studies in Epidemiology.

### Search Strategy

A systematic search for studies was performed using online international databases, namely, Web of Science,

Science Direct, PubMed, Scopus, and Google scholar, to determine suitable studies published from 2000 to 2021. The references of these studies were examined to improve search sensitivity. The following search terms were used in combination for search strategies: “Semen,” “Infertility men,” “Iran,” “Virus,” and “Bacteria,” which were combined with and/or not.

### Study Selection

All full texts or abstracts of the literature were excluded from the database search and reviewed on the advanced search. First, after limiting the search, non-relevant and duplicate studies were excluded. Then, studies were screened after checking the titles, abstracts, and full texts. Later, appropriate studies were included in our study.

This study aimed to estimate the prevalence of viral and bacterial agents in the semen of infertile men and to analyze the relationship between bacterial and viral agents and infertility in men; the inclusion and exclusion criteria are as follows.

### Inclusion Criteria

In this study, (PICOS) search strategy for descriptive studies aimed to determine infertile men (P) and the prevalence of viral and bacterial agents in descriptive studies (S). For analytical studies, PICOS was used to identify infertile men (P), viral and bacterial agents (E), fertile men (C), odds ratio of viral and bacterial agents in the semen of infertile men to fertile (O), and case-control studies (S).

All studies that passed the above assessment phases for high-quality scores were selected if they met the following conditions: (1) case-control studies on associations between bacterial infections and infertility, (2) case-control studies on associations between viral infections and infertility, (3) cross-sectional studies based on the prevalence of bacterial infections in the semen of infertile men, (4) cross-sectional studies based on the prevalence of viral infections in the semen of infertile men, and (5) both English and Persian studies.

### Exclusion Criteria

The following types of studies were excluded: (1) case reports or case series articles, (2) articles with no complete access to full text, (3) duplicate studies, (4) conference abstracts without full-text letters or review studies, (5) studies with low quality, and (6) studies published in languages other than English and Persian.

### Quality Assessment

The quality of this meta-analysis was determined following the Newcastle-Ottawa Scale (NOS) statement. The NOS checklist covers methodology, comparability, and outcome. Depending on the quality of the analysis, the studies were divided into three groups: good quality, fair quality, and poor quality.

### Data Extraction

After selection of appropriate literature, the following data were extracted: authors, publication year, geographical regions, publication language, type of study, number of infertile men infected with viral/or bacterial agents, sample size, agent, and source of the sample. Data were extracted and entered into a Microsoft Excel spreadsheet.

### Statistical Analysis

In this study, Stata ver. 14 package (StataCorp, College Station, TX, United States) was used for data analysis. A contingency table was formed for each case-control study for the case and control groups. Data were weighted and combined using the inverse variance method. The heterogeneity index (*I*^2^) between studies was determined by Cochran (*Q*) and *I*^2^ tests. Also, according to Higgins and Thompson (15), an *I*^2^ value of less than 25% indicates low heterogeneity, 25–75% indicates moderate heterogeneity, and over 75% indicates high heterogeneity. A random-effects model was used to estimate the odds ratio of male infertility in the case group and the control group and to estimate the frequency of viruses or bacteria in the semen of infertile men. Odds ratios were calculated with 95% CIs on forest plots. In this diagram, the square size shows the weight of each study, and related lines on either side represent a 95% CI. A meta-regression test was performed to evaluate the effect of microorganisms on heterogeneity. An Egger’s test was conducted and a funnel plot chart with a significant level of less than 0.1 was used to evaluate publication bias. In addition, a sensitivity analysis was performed to investigate the effect of each early study on the overall pooled odds ratio.

## Results

**FIGURE 1:**
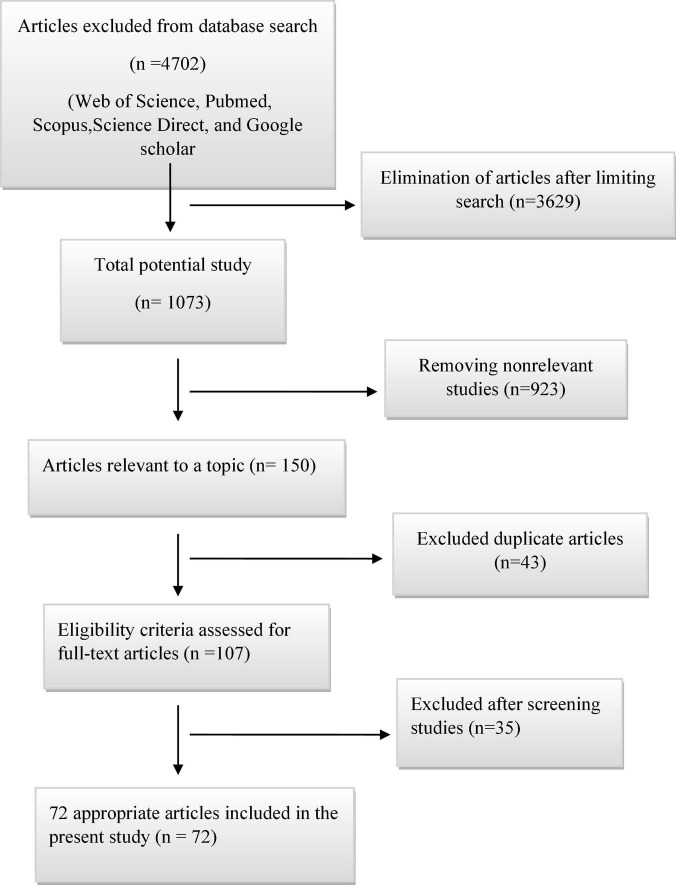
Flowchart of primary studies included in the meta-analysis.

**FIGURE 2:**
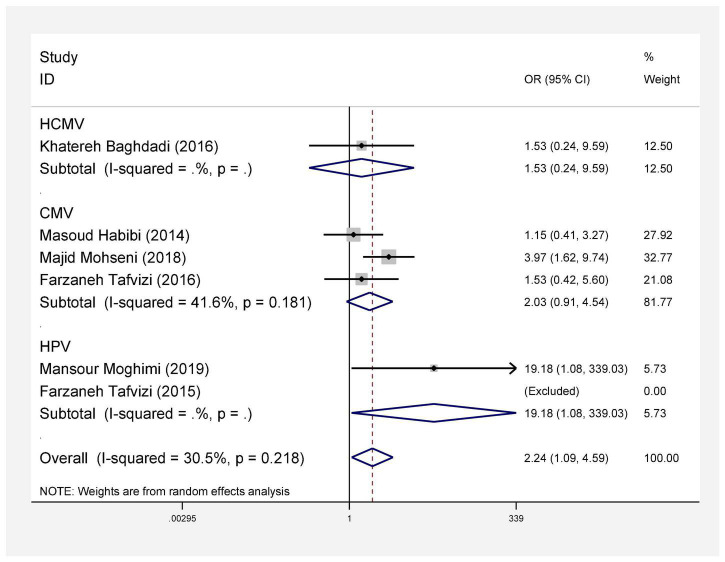
Estimation of odds ratio of male infertility due to exposure to viruses.

**FIGURE 3:**
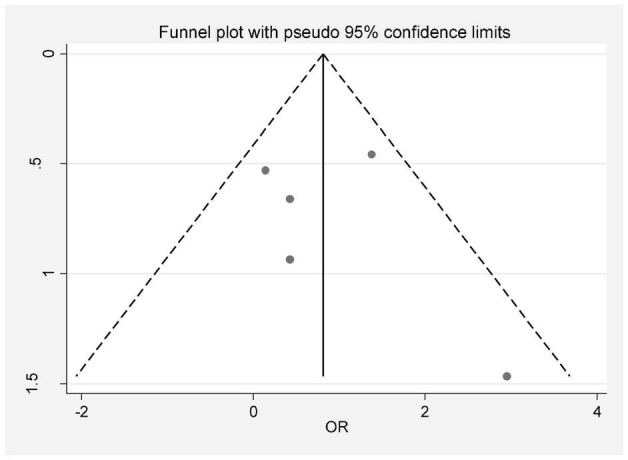
Publication bias based on a funnel plot.

**FIGURE 4:**
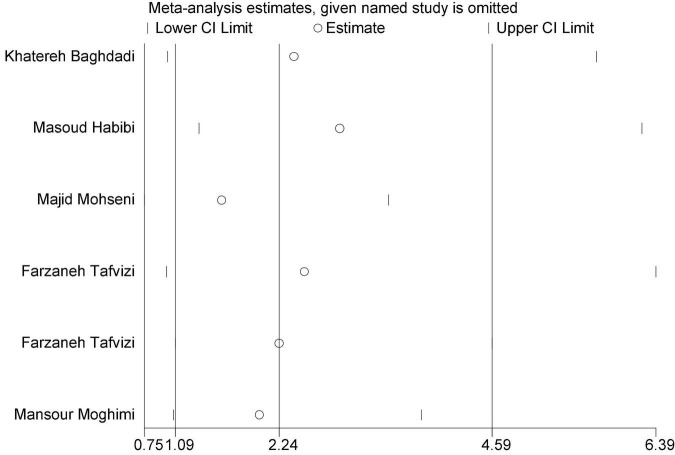
Sensitivity analysis of the primary studies included in the meta-analysis.

**FIGURE 5:**
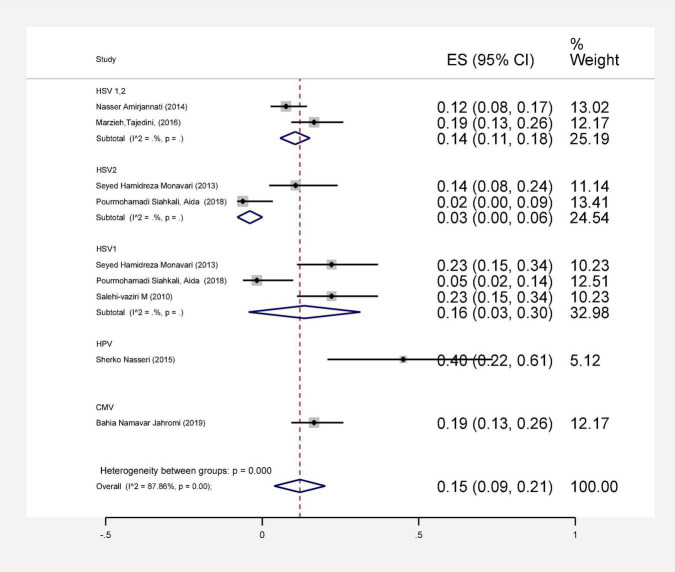
Estimating the prevalence of viral agents in each of the initial studies and pooled estimate with a 95% confidence interval.

**FIGURE 6:**
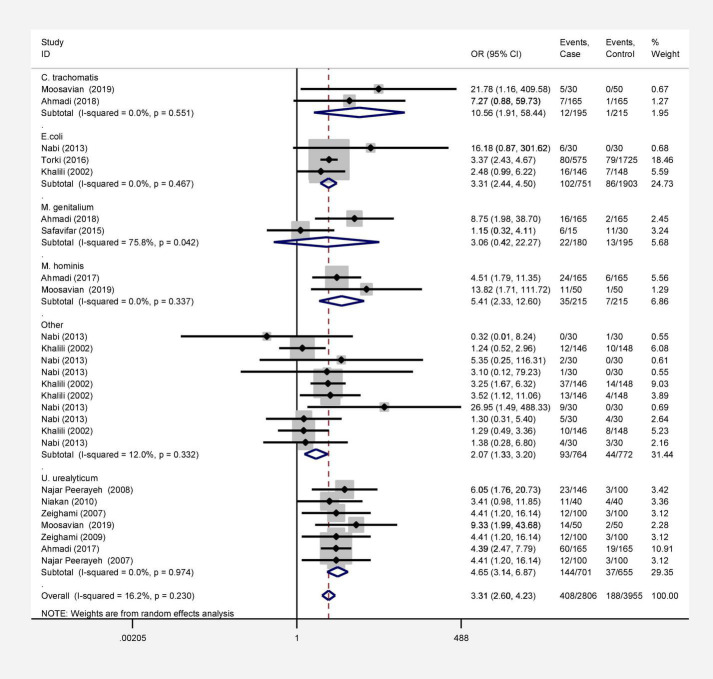
Estimation of odds ratio of male infertility due to exposure to different bacteria.

**FIGURE 7:**
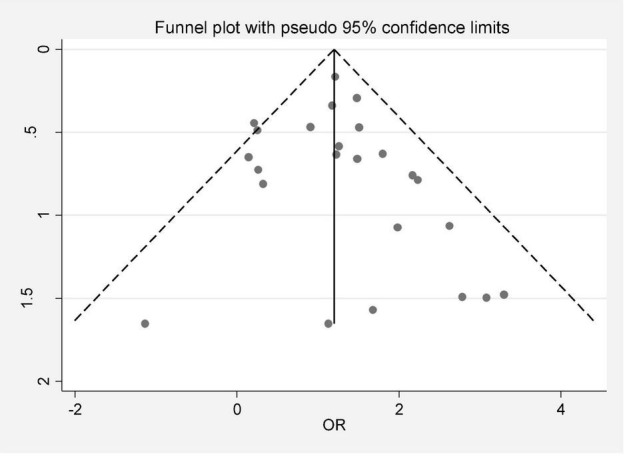
Publication bias (odds ratio of male infertility due to exposure to different bacteria based on the funnel plot).

**FIGURE 8:**
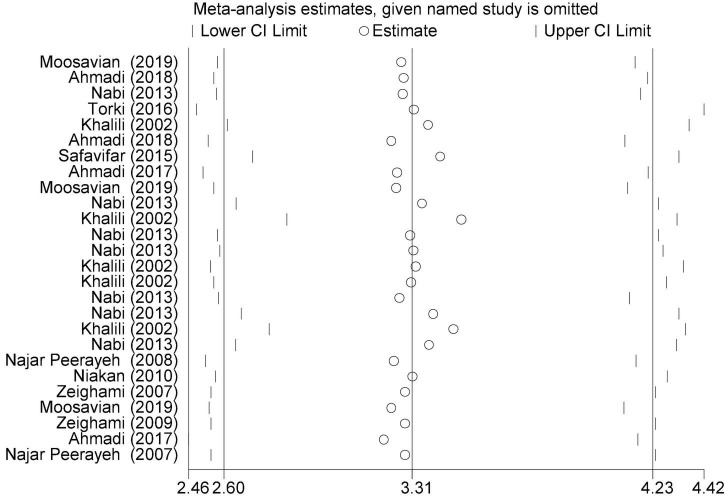
Sensitivity analysis (overall estimation of the odds ratio of male infertility due to exposure to different bacteria).

**FIGURE 9:**
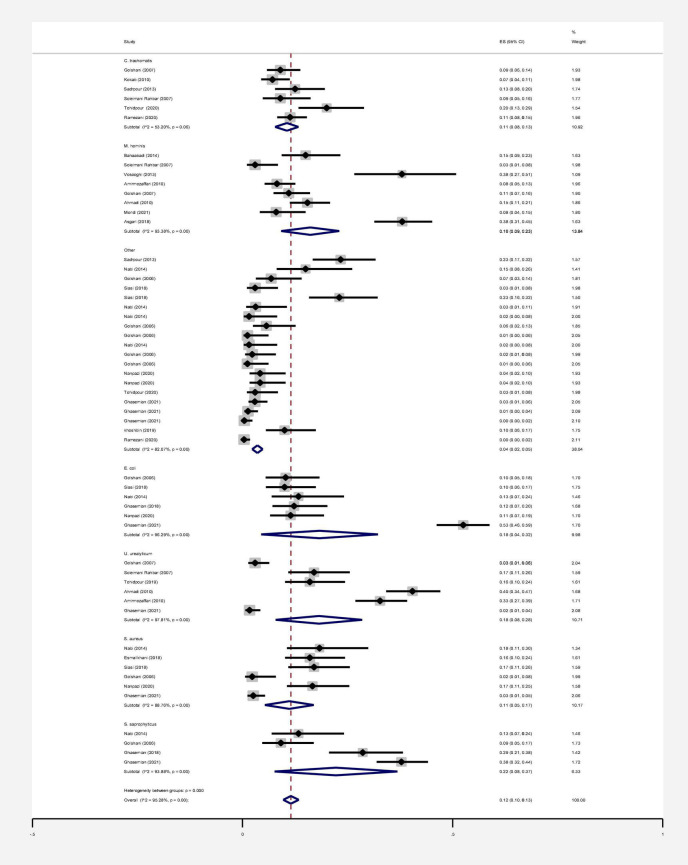
Estimating the prevalence of bacterial agents in each of the initial studies and pooled estimate with a 95% confidence interval.

In this review, a total of 4,702 articles were found from the online databases (Web of Science, PubMed, Scopus, Science Direct, and Google scholar). After limiting the search strategy, the studies were restricted to 1,073. A total of 923 non-relevant studies were then excluded. After removing 43 duplicate studies, 107 articles were assessed for eligibility criteria. Finally, 72 appropriate studies were included in this meta-analysis (Figure 1 and Tables 1–4). The relationship between human cytomegalovirus (HCMV), cytomegalovirus (CMV), and human papillomavirus (HPV) and male infertility was investigated in six case-control studies. The heterogeneity among the primary studies was not significant (*I*^2^: 30.5%, Q: 5.8, *P*: 0.218). By combining the results of primary studies based on the random effect model, the odds ratio of male infertility in men exposed to HCMV, CMV, and HPV was higher than that in men without exposure to these viruses 2.24 (1.09–4.59) 95%. This difference was statistically significant. It should be noted that, in Tafvizi’s (16) study, the frequency of HPV was zero in the case and control groups (Figure 2). A funnel plot is used to investigate publication bias to estimate the odds ratio of male infertility due to exposure to CMV, HCMV, and HPV. According to the funnel plot and Egger test, there is no publication bias (β = 0.74, *P* = 0.698). Also, the results of the meta-regression test show that the virus type did not affect the heterogeneity and diversity of the results (β = 0.96, *P* = 0.351 (Figure 3). The sensitivity analysis results showed that the effect of each primary study on the overall estimation of the odds ratio of male infertility due to exposure to CMV, HCMV, and HPV was not significant (Figure 4). The heterogeneity of 9 studies in estimating the frequency of the HPV, herpes simplex virus 1 (HSV1), herpes simplex virus 2 (HSV2), and herpes simplex virus 1-2 (HSV1-2) is high (*I*^2^: 87.86%, Q: 65.9, *P* < 0.001). By combining the results of eight primary studies, the overall incidence of these viruses in infertile men was 15% (95% CI: 9–21 (Figure 5). A total of 26 case-control studies examined the association between *Ureaplasma urealyticum, Mycoplasma hominis, Mycoplasma genitalium, Escherichia coli, Chlamydia trachomatis, Streptococcus pyogenes, Staphylococcus aureus, Staphylococcus saprophyticus, Staphylococcus epidermidis, and* other bacterial exposure and male infertility. The heterogeneity among the primary studies was not significant (*I*^2^: 10.2%, *Q*: 29.85, *P* = 0.23). The statistical significance of the results shows that the odds ratio of male infertility in men exposed to the bacteria was higher than that in men without exposure to these bacteria (3.15; 95% CI: 2.60–4.23) (Figure 6). A funnel plot was used to determine publication bias in estimating the odds ratio of male infertility due to exposure to different bacteria. According to the funnel plot and Egger test, there is no publication bias (β = 0.31, *P* = 0.431) (Figure 7). The sensitivity analysis showed that the effect of each primary study on the overall estimation of the odds ratio of male infertility due to exposure to different bacteria was not significant (Figure 8). The result of the study for estimating the frequency of bacteria showed significant heterogeneity among the results of 56 primary studies (*I*^2^: 95.3%, *Q*: 1,166.1, *P* < 0.001), and the overall incidence of bacteria in infertile men was 12% (95% CI: 10–13) (Figure 9).

**TABLE 1:** Case-control virus studies included in the meta-analysis.

References	Publication language	Case (N)		Control (N)		Agent	Source of sample	OR (95%)
		Event	Total	Event	Total			
Baghdadi et al. (17)	English	3	50	2	50	HCMV	Semen	1.53 (0.24, 9.59)
Habibi et al. (18)	English	20	154	5	46	CMV	Semen	1.15 (0.41, 3.27)
Mohseni et al. (19)	English	23	100	7	100	CMV	Semen	3.97 (1.62, 9.74)
Tafvizi et al. (20)	Persian	6	100	4	100	CMV	Semen	1.53 (0.42, 5.60)
Tafvizi et al. (16)	English	0	50	0	50	HPV	Semen	Excluded
Moghimi et al. (21)	English	8	70	0	70	HPV	Semen	19.18 (1.08, 339.03)
Pooled estimate (random model)		60	524	18	416	2.24 (1.09, 4.59)		

**TABLE 2:** Cross-sectional virus studies included in the meta-analysis.

References	Publication language	Number of virus infection	Sample size	Agent	Source of sample	Percentage of virus infection (%)
Tajedini et al. (22)	English	28	150	HSV1-2	Semen	0.19 (0.13, 0.26)
Pourmohamadi and Amini (23)	Persian	3	60	HSV1	Semen	0.05 (0.02, 0.14)
Pourmohamadi and Amini (23)	Persian	1	60	HSV2	Semen	0.02 (0.00, 0.09)
Salehi-vaziri et al. (24)	English	16	70	HSV1	Semen	0.23 (0.03, 0.30)
Monavari et al. (25)	English	10	70	HSV2	Semen	0.14 (0.08, 0.24)
Monavari et al. (25)	English	16	70	HSV1	Semen	0.23 (0.15, 0.34)
Amirjannati et al. (26)	English	26	217	HSV1-2	Semen	0.12 (0.08, 0.17)
Nasseri et al. (27)	English	8	20	HPV	Semen	0.40 (0.22, 0.61)
Jahromi et al. (28)	English	28	150	CMV	Semen	0.19 (0.13, 0.26)
Pooled estimate (random model)		136	867	0.15 (0.09, 0.21)		
						

**TABLE 3:** Case-control studies on bacteria included in the meta-analysis.

References	Publication language	Case (N)		Control (N)		Agent	Source of sample	OR (95%)
		Event	Total	Event	Total			
Zeighami et al. (29)	English	12	100	3	100	*U. urealyticum*	Semen	4.41 (1.20, 16.14)
Ahmadi et al. (30)	English	60	165	19	165	*U. urealyticum*	Semen	4.39 (2.47, 7.79)
Peerayeh et al. (31)	English	23	146	3	100	*U. urealyticum*	Semen	6.05 (1.76, 20.73)
Zeighami et al. (32)	English	12	100	3	100	*U. urealyticum*	Semen	4.41 (1.20, 16.14)
Zeighami et al. (29)	English	12	100	3	100	*U. urealyticum*	Semen	4.41 (1.20, 16.14)
Niakan et al. (33)	English	11	40	4	40	*U. urealyticum*	Semen	3.41 (0.98, 11.85)
Ahmadi et al. (34)	English	24	165	6	165	*M. hominis*	Semen	4.51 (1.79, 11.35)
Ahmadi et al. (35)	English	16	165	2	165	*M. genitalium*	Semen	8.75 (1.98, 38.70)
Safavifar et al. (36)	English	6	15	11	30	*M. genitalium*	Semen	1.15 (0.32, 4.11)
Torki et al. (37)	English	80	575	79	1725	*E. coli*	Semen	3.37 (2.43, 4.67)
Khalili et al. (38)	English	16	146	7	148	*E. coli*	Semen	2.48 (0.99, 6.22)
Nabi et al. (39)	English	6	30	0	30	*E. coli*	Semen	16.18 (0.87, 301.62)
Ahmadi et al. (40)	English	7	165	1	165	*C. trachomatis*	Semen	7.27 (0.88, 59.73)
Khalili et al. (38)	English	10	146	8	148	*S. pyogenes*	Semen	1.29 (0.49, 3.36)
Nabi et al. (39)	English	9	30	0	30	*S. aureus*	Semen	26.95 (1.49, 488.33)
Nabi et al. (39)	English	4	30	3	30	*S. saprophyticus*	Semen	1.38 (0.28, 6.80)
Nabi et al. (39)	English	5	30	4	30	*S. epidermidis*	Semen	1.30 (0.31, 5.40)
Khalili et al. (38)	English	37	146	14	148	*Other*	Semen	3.25 (1.67, 6.32)
Nabi et al. (39)	English	0	30	1	30	*Other*	Semen	0.32 (0.01, 8.24)
Khalili et al. (38)	English	12	146	10	148	*Other*	Semen	1.24 (0.52, 2.96)
Khalili et al. (38)	English	13	146	4	148	*Other*	Semen	3.52 (1.12, 11.06)
Nabi et al. (39)	English	2	30	0	30	*Other*	Semen	5.35 (0.25, 79.23)
Nabi et al. (39)	English	1	30	0	30	*Other*	Semen	3.10 (0.12, 79.23)
Moosavian et al. (41)	English	5	50	0	50	*C. trachomatis*	Semen	7.27 (1.16, 409.58)
Moosavian et al. (41)	English	14	50	2	50	*U. urealyticum*	Semen	9.33 (1.99, 43.68)
Moosavian et al. (41)	English	11	50	1	50	*M. hominis*	Semen	3.10 (0.12, 79.23)
Pooled estimate (random model)		408	2,826	187	3,955	3.31 (2.60–4.23)		

**TABLE 4:** Cross-sectional studies on bacteria included in the meta-analysis.

References	Publication language	Number of bacterial infection	Sample size	Agent	Source of sample	Percentage of virus infection (%)
Golshani et al. (42)	English	18	200	*C. trachomatis*	Semen	0.09 (0.06, 0.14)
Kokab et al. (43)	English	16	255	*C. trachomatis*	Semen	0.07 (0.04, 0.11)
Sadrpour et al. (44)	English	15	120	*C. trachomatis*	Semen	0.13 (0.08, 0.20)
Soleimani Rahbar et al. (45)	Persian	9	100	*C. trachomatis*	Semen	0.09 (0.05, 0.16)
Bahaabadi et al. (46)	English	15	100	*M. hominis*	Semen	0.15 (0.09, 0.23)
Soleimani Rahbar et al. (45)	English	3	100	*M. hominis*	Semen	0.03 (0.01, 0.08)
Vosooghi et al. (47)	English	22	58	*M. hominis*	Semen	0.38 (0.27, 0.51)
Amirmozaffari et al. (48)	English	18	220	*M. hominis*	Semen	0.08 (0.05, 0.13)
Golshani et al. (42)	English	22	200	*M. hominis*	Semen	0.11 (0.07, 0.16)
Ahmadi et al. (49)	English	34	220	*M. hominis*	Semen	0.15 (0.11, 0.21)
Sadrpour et al. (44)	English	28	120	*M. genitalium*	Semen	0.23 (0.17, 0.32)
Golshani et al. (50)	English	9	88	*E. coli*	Semen	0.10 (0.05, 0.18)
Siasi et al. (51)	English	10	100	*E. coli*	Semen	0.10 (0.06, 0.17)
Nabi et al. (52)	English	8	65	*E. coli*	Semen	0.13 (0.07, 0.24)
Ghasemian et al. (53)	English	12	98	*E. coli*	Semen	0.12 (0.07, 0.20)
Golshani et al. (42)	English	6	200	*U. urealyticum*	Semen	0.03 (0.01, 0.06)
Soleimani Rahbar et al. (45)	English	17	100	*U. urealyticum*	Semen	0.17 (0.11, 0.26)
Tohidpour et al. (54)	English	16	100	*U. urealyticum*	Semen	0.16 (0.10, 0.24)
Ahmadi et al. (49)	English	89	220	*U. urealyticum*	Semen	0.40 (0.34, 0.47)
Amirmozaffari et al. (48)	English	72	220	*U. urealyticum*	Semen	0.33 (0.27, 0.39)
Nabi et al. (52)	English	11	65	*S. aureus*	Semen	0.18 (0.11, 0.30)
Esmailkhani et al. (55)	English	16	100	*S. aureus*	Semen	0.16 (0.10, 0.24)
Siasi et al. (51)	English	17	100	*S. aureus*	Semen	0.17 (0.11, 0.26)
Golshani et al. (50)	English	2	88	*S. aureus*	Semen	0.02 (0.01, 0.08)
Nabi et al. (52)	English	8	65	*S. saprophyticus*	Semen	0.13 (0.07, 0.24)
Golshani et al. (50)	English	8	88	*S. saprophyticus*	Semen	0.09 (0.05, 0.17)
Ghasemian et al. (53)	English	28	98	*S. saprophyticus*	Semen	0.29 (0.21, 0.38)
Nabi et al. (52)	English	9	65	*S. epidermidis*	Semen	0.15 (0.08, 0.26)
Golshani et al. (50)	English	6	88	*S. agalactiae*	Semen	0.07 (0.03, 0.14)
Siasi et al. (51)	English	3	100	*K. pneumoniae*	Semen	0.03 (0.01, 0.08)
Siasi et al. (51)	English	23	100	*Other*	Semen	0.23 (0.16, 0.32)
Nabi et al. (52)	English	2	65	*Other*	Semen	0.03 (0.01, 0.11)
Nabi et al. (52)	English	1	65	*Other*	Semen	0.02 (0.00, 0.08)
Golshani et al. (50)	English	5	88	*Other*	Semen	0.06 (0.02, 0.13)
Golshani et al. (50)	English	1	88	*Other*	Semen	0.01 (0.00, 0.06)
Nabi et al. (54)	English	1	65	*Other*	Semen	0.02 (0.01, 0.08)
Golshani et al. (50)	English	2	88	*Other*	Semen	0.02 (0.0, 0.08)
Golshani et al. (50)	English	1	88	*Other*	Semen	0.01 (0.00, 0.06)
Moridi et al. (56)	English	8	100	*M. hominis*	Semen	0.08 (0.04, 0.15)
Nanpazi et al. (57)	English	16	96	*S. aureus*	Semen	0.17 (0.11, 0.25)
Nanpazi et al. (57)	English	11	96	*E. coli*	Semen	0.11 (0.07, 0.19)
Nanpazi et al. (57)	English	4	96	*Klebsiella*	Semen	0.04 (0.02, 0.10)
Nanpazi et al. (57)	English	4	96	*S. epidermidis*	Semen	0.04 (0.02, 0.10)
Asgari et al. (58)	Persian	71	187	*M. hominis*	Semen	0.38 (0.31, 0.45)
Tohidpour et al. (59)	English	20	100	*C. trachomatis*	Semen	0.20 (0.13, 0.29)
Tohidpour et al. (59)	English	3	100	*L. monocytogenes*	Semen	0.03 (0.01, 0.08)
Ghasemian et al. (60)	English	125	238	*E. coli*	Semen	0.53 (0.46, 0.59)
Ghasemian et al. (60)	English	90	238	*S. saprophyticus*	Semen	0.38 (0.32, 0.44)
Ghasemian et al. (60)	English	7	238	*E. faecalis*	Semen	0.03 (0.01, 0.06)
Ghasemian et al. (60)	English	6	238	*S. aureus*	Semen	0.03 (0.01, 0.05)
Ghasemian et al. (60)	English	4	238	*U. urealyticum*	Semen	0.02 (0.01, 0.04)
Ghasemian et al. (60)	English	3	238	*S. agalactiae*	Semen	0.01 (0.00, 0.02)
Ghasemian et al. (60)	English	1	238	*G. vaginalis*	Semen	0.00 (0.00, 0.02)
Paknejadi (61)	Persian	10	100	*H. pylori*	Semen	0.10 (0.06, 0.17)
Ramezani et al. (62)	English	35	309	*C. trachomatis*	Semen	0.11 (0.08, 0.13)
Ramezani et al. (62)	English	1	309	*N. gonorrhoeae*	Semen	0.00 (0.00, 0.02)
Pooled estimate (random model)		1,002	7,593	0.04 (0.02, 0.05)		

## Discussion

Infertility affects about 15% of couples globally and about half of these cases are because of male factors. Major etiological variants include microbial infectious and inflammatory disorders in the reproductive system. Male infertility can be caused by various microorganisms, although the direct effects of bacterial and viral infections on male infertility are still debated (63, 64). To date, there has been no systematic review and meta-analysis study on the frequency and role of viral and bacterial infections simultaneously in infertile men in Iran. Based on our results, all 9 studies investigating the prevalence of HPV, HSV1, HSV2, and HSV1-2 in infertile men have a frequency of 15% (CI 95%: 9–21). The association between HCMV, CMV, and HPV and male infertility was evaluated in 6 case-control studies. The ratio of male infertility due to these viruses (1.09–4.59) was 2.24 times higher than those without viruses. The association between HPV infection and male infertility has also been recently explored, and the available data are controversial (65, 66). In a cross-sectional clinical study, Foresta et al. (67) have also shown that sperm motility in patients positive for HPV was considerably reduced (67). Perino et al. indicated that HPV infection was associated with infertility in couples. These presented a diminished pregnancy rate and an elevated abortion frequency in couples infected with HPV than those not infected (68). Male infertility and aberrant sperm parameters are linked to HSV infection, which disrupts the male accessory genital system (69, 70). HSV infections in sperm vary greatly among investigations (71). Kurscheidt et al. have previously reported that HSV infections in male partners of infertile couples could cause alterations in spermatozoa and seminal fluid, affecting fertility (72). This analysis is consistent with a previous report by Bezold et al. (69). They found that HSV DNA-positive semen specimens had meaningfully decreased sperm count and motility. Based on available data, the occurrence of HCMV DNA in the semen of fertile and infertile men with seropositive HCMV is between 8 and 65% (73). However, HCMV-caused male infertility is commonly unusual (74, 75). Pallier et al. have previously reported that HCMV particles in the semen are not correlated with sperm motility (76). Most of the evaluated articles in our study were about viruses that were possibly associated with male infertility. In line with studies conducted on viral infection, numerous efforts have been directed toward identifying the role of bacterial infections in male infertility. Bacterial infection was considered a significant element of infertility in the semen of asymptomatic infertile men (35). In a recent study, among a total of 50 infertile male semen samples, 45 (90%) were infected with at least one type of bacterial strain. In comparison, in five samples of infertile semen, microorganisms were not detected. The most common bacterial genus was *Enterococcus* (32%), followed by *Klebsiella* (22%) (77). In another study, Domes et al. reported 15% bacteriospermia in a male factor infertility population (1,200/7,852), including 22 bacterial species. Among positive cultures, *Enterococcus faecalis* was the most frequent, with an occurrence of 56% (15/60) (78). In 56 studies that entered our meta-analysis, the pooled prevalence of bacterial infections in the semen of infertile men was 12% (95% CI 10–13%). The relationship between infertility in men and bacteria was evaluated in 26 case-control studies. The results show that the ratio of chance of infertility in men exposed to bacteria with a 95% confidence interval is equal to 2.60–4.23, which is 3.31 times that of people without bacteria. Moreover, Zeyad et al. showed that bacterial infections have significant negative effects on sperm parameters. Sperm concentration, motility, and progression, and chromatin condensation were significantly lower in infected patients (79).

## Limitation

There are some limitations in this meta-analysis:

• High heterogeneity among the results of preliminary studies, which included study population, methodology of studies, sampling, and semen analysis method in infertile males.
• Lack of socioeconomic and demographic information of infertile men may have led to the heterogeneity.
• Different criteria for the screening and recruitment of infertile men in the included studies.

## Conclusion

The presence of viral and bacterial infections is a risk factor and could impair male fertility potential by decreasing sperm quality. The current systematic review and meta-analysis provides the overall bacterial and viral infection frequency in infertile men and data on etiologic agents of viruses and bacteria in Iran. Also, this meta-analysis supports the hypothesis that co-infection could play a key role in impairing sperm quality, motility, and mobility in the male population.

## Data Availability Statement

The original contributions presented in the study are included in the article/Supplementary Material, further inquiries can be directed to the corresponding author/s.

## Author Contributions

TM and MG conceived and designed the study, performed the literature search, and collected the data. HJ and MH performed the literature review. MM performed the statistical analysis. All authors read and approved the final version of the manuscript.

## Conflict of Interest

The authors declare that the research was conducted in the absence of any commercial or financial relationships that could be construed as a potential conflict of interest.

## Publisher’s Note

All claims expressed in this article are solely those of the authors and do not necessarily represent those of their affiliated organizations, or those of the publisher, the editors and the reviewers. Any product that may be evaluated in this article, or claim that may be made by its manufacturer, is not guaranteed or endorsed by the publisher.
